# Resetting the Respiratory Rhythm with a Spinal Central Pattern Generator

**DOI:** 10.1523/ENEURO.0116-19.2019

**Published:** 2019-04-30

**Authors:** Roberto Meza, Nayeli Huidobro, Mayra Moreno-Castillo, Abraham Mendez-Fernandez, Jorge Flores-Hernandez, Amira Flores, Elias Manjarrez

**Affiliations:** Instituto de Fisiología, Benemérita Universidad Autónoma de Puebla, Puebla, México, 72570

**Keywords:** cat, phase resetting, resetting, respiratory, scratching CPG, spinal cord

## Abstract

There is evidence that a variety of central and afferent stimuli, including swallowing, can produce phase resetting in the respiratory rhythmicity. Also, there are reports about the intrinsic linkage between locomotion and respiration. However, little is known about the interaction between the central pattern generators (CPGs) for scratching and respiration. The present study aims to examine whether the activation of scratching CPG produces phase resetting of the respiratory rhythm. We employed decerebrate cats to apply brief tactile stimuli to the pinna during the inspiratory-expiratory transition. We observed that those stimuli to the pinna not eliciting fictive scratching did not reset the respiratory rhythm. However, when the pinna stimuli elicited fictive scratching, then the respiratory rhythm exhibited a significant phase resetting. We also found interneurons in the medulla oblongata exhibiting phase resetting related to scratching-CPG episodes. This second finding suggests that this type of resetting involves brainstem components of the respiratory CPG. These results shed new light on the resetting action from a spinal CPG on the respiratory rhythm.

## Significance Statement

Here, we report for the first time the resetting influence of the spinal central pattern generator (CPG) for scratching on the respiratory rhythm. We conclude that fictive scratching, as a “central stimulus” delivered to the respiratory CPG, can produce phase resetting in phrenic nerve activity and the firing activity of interneurons from the medulla oblongata in decerebrate paralyzed cats.

## Introduction

Phase resetting is the transient interruption of activity of an oscillator by a precisely timed perturbation, which prevents the completion of a cycle and restarts it (Winfree, 1987). This phenomenon is widespread in the central nervous system, and there is evidence that neurons interact through phase resetting to establish a time frame for information encoding and decoding (Canavier, 2015), among other behaviors. In particular, the analysis of phase resetting is of interest in circuits known as central pattern generators (CPGs) because the elucidation of their structure and organization is a work in progress and because the cooperation between CPGs is poorly understood (Berg et al., 2007; Stuart and Hultborn, 2008; McCrea and Rybak, 2008; Harris-Warrick, 2010; Enriquez-Denton et al., 2012; Tsuruyama et al., 2013; Grillner and El Manira, 2015; Kiehn, 2016; Ramirez et al., 2016; Pujala et al., 2016; Berg, 2017; Stein, 2018; Steuer and Guertin, 2019).

Studies performed on the respiratory CPG have shown that the normal coordination of different physiological functions of the upper airways (such as swallowing) involves phase resetting, as opposed to a mere interruption of the respiratory rhythm (Paydarfar et al., 1986, 1995, 1998; Paydarfar and Eldridge, 1987; McFarland and Lund, 1995). For instance, electrical stimulation of the midbrain reticular formation and periaqueductal gray matter produces both a phase resetting and a facilitation of the respiratory rhythm in cats (Paydarfar and Eldridge, 1987). Furthermore, stimulation of the nasal mucosa, the carotid sinus and the radial nerve (Kawahara et al., 1988) can elicit a response on the respiratory oscillator. Studies also exist correlating specific neuronal types in the pre-Bötzinger complex (pre-BötC) and the perturbations in their cycles to specific behaviors like vomiting in decerebrate dogs (Fukuda and Tomoshige, 1997). On the other hand, several studies have shown entrainment of the respiratory rhythm by activation of the locomotor-CPG or related afferents both *in vivo* (Viala and Freton, 1983; Viala et al., 1987) and *in vitro* (Kawahara et al., 1988; Le Gal et al., 2014) in various animal models and humans (Bramble and Carrier, 1983).

In summary, there is evidence of a phase-resetting relationship between the respiratory CPG with other CPGs that coexist around the same gross anatomic regions, as well as an entrainment relationship with the CPG for locomotion. However, to our knowledge, there are no studies showing the resetting action of the scratching CPG on the respiratory rhythm, even that there is a pioneering report illustrating the relationships between the respiratory rhythm and scratching (King, 1931). Our study aims to show that the activation of a spinal CPG can reset the respiratory rhythmicity of the phrenic nerve and single neurons from a brainstem respiratory center. The advantage of our approach using the scratching CPG to reset the respiratory rhythm instead of the locomotion CPG is that the scratching episodes are more easily produced than the locomotion episodes, and just a brief mechanical stimulation to the pinna can be enough to produce scratching.

The indication of the fact that there is an activating system for fictive scratching around the bulbar reticular formation makes the possibility of physiological resetting interaction from the scratching CPG and the respiration CPG worthy of pursuit (Tapia et al., 2013). The location of this reticular activating system could suggest an overlap or interaction with relevant structures for respiration, as the nucleus tractus solitarius, or the ventral respiratory column. This would guarantee the adequate interaction between non-overlapping and functionally distinct CPGs, necessary for the animal’s safeguarding. In particular, here we examine which type of resetting produces the scratching CPG on the respiratory CPG. Paydarfar et al. (1986) and Paydarfar and Eldridge (1987) described three types of resetting: type 1, type 0 and “ambiguous.” The type 1, is a resetting in which a weak stimuli causes little phase resetting, and the latency from stimulus to subsequent cycles (called the cophase) falls by one cycle as the stimulus time (called the old phase) is advanced through the entire cycle. The type 0, is a resetting in which a strong stimulus causes large perturbations of phrenic nerve rhythm, and there is no net change in the cophase as the old phase increases one full cycle. The ambiguous is a resetting in which a stimulus with moderate strength produces a phase singularity, recorded as highly variable latencies to the next cycles for stimuli given at a single old phase (i.e., there is unpredictable resetting with stimuli given only at a specific old phase). The present study constitutes the first approach to identify whether scratching, as a resetting “stimulus” delivered to the respiratory CPG, can produce phase resetting in phrenic nerve activity or brainstem interneurons in decerebrate/paralyzed cats.

## Materials and Methods

### Animal preparation

We performed experiments in four adult cats (2.3–3.7 kg, two males and two females). We followed similar experimental procedures as in previous reports from our group (Cuellar et al., 2009, 2018; Pérez et al., 2009; Tapia et al., 2013). During surgery, we induced and maintained deep anesthesia with isoflurane at 2%. At the beginning of any surgical procedure, we administered atropine (0.05 mg/kg) and dexamethasone (2 mg/kg). The protocol was approved by the ethics committee (CICUAL-Proyecto-00489) of the Benemérita Universidad Autónoma de Puebla. We followed the guidelines contained in the National Institutes of Health Guide for the Care and Use of Laboratory Animals (publication 85-23, revised in 1985) and the Mexican regulations (NOM-062-ZOO-1999). We verified the level of anesthesia by monitoring the arterial blood pressure from the carotid artery and by testing for the lack of withdrawal reflexes and muscle tone. We administered a mix of bicarbonate (100 mM) and glucose (5%) solution at a rate of 5 ml/h through the radial vein during all the experiment.

For recordings, we dissected the bilateral tibialis anterior (TA) and the medial gastrocnemius (MG) nerves. The lumbosacral and cervical spinal segments were exposed, and the dura mater was removed. We dissected the C5 root of the phrenic nerve (Eldridge et al., 1989). After these surgical procedures, the animal was mounted on a stereotaxic apparatus. Pools were formed with the skin around the exposed tissues and filled with mineral oil (after placement of the electrodes) and maintained at a constant temperature (37°C). The decerebration consisted of a mechanical precollicular–postmamillary transection with the removal of both cortices and all tissue rostral to the transection. We applied SURGICEL Absorbable Hemostat on the exposed neural tissues, and the empty cavity was filled with Agar-Agar. We discontinued the anesthesia 3 min after the decerebration. We administered dextran and saline solutions as necessary to maintain blood pressure between 80 and 120 mmHg. Then the animals were paralyzed with pancuronium bromide (Pavulon; Organon) and artificially ventilated. At the end of the experiment, each animal was euthanized with an overdose of pentobarbital. We applied D-tubocurarine (0.1%) on the surface of the C1–C2 segments to induce fictive scratching after mechanical stimulation of the pinna (Feldberg and Fleischauer, 1960). Scratching was produced by brief (Tapia et al., 2013) mechanical stimulation of scratch reflex receptive fields located in the left pinna.

### Recording

We recorded electroneurograms with conventional hook electrodes from the distal end of the left flexor TA, and extensor MG sectioned nerves, as well as the electrical activity from the left C5 root of the phrenic nerve. An electret microphone was employed to record the sounds of respiration in the artificially ventilated cats. We employed Grass-P511 AC amplifiers (Astromed) with a 0.05- to 30-kHz bandpass filter and the Digidata system (Molecular Devices) with a sampling rate of 50 kHz.

In two experiments, we used quartz/platinum-tungsten fiber electrodes to record multiunit neuronal activity (impedance 5–7 MOhm) with a five-channel Minimatrix system (Thomas Recording). We obtained recordings of neurons from the medulla oblongata, and we performed a histological reconstruction of recording sites.

### Spike sorting analysis

We used the unsupervised spike-sorting software “waveclus” developed by Quiroga et al. (2004) to classify unitary spikes from the multiunit recordings. We obtained raster displays of firing activity of medullary neurons following the respiratory rhythm, before, during, and after the scratching episodes.

### Descriptive statistical analysis

Correlations were tested with the nonparametric Spearman’s rank coefficient method (*p* < 0.001). Data are indicated as mean ± standard deviation.

### Random experimental paradigm

**Figure 1.**
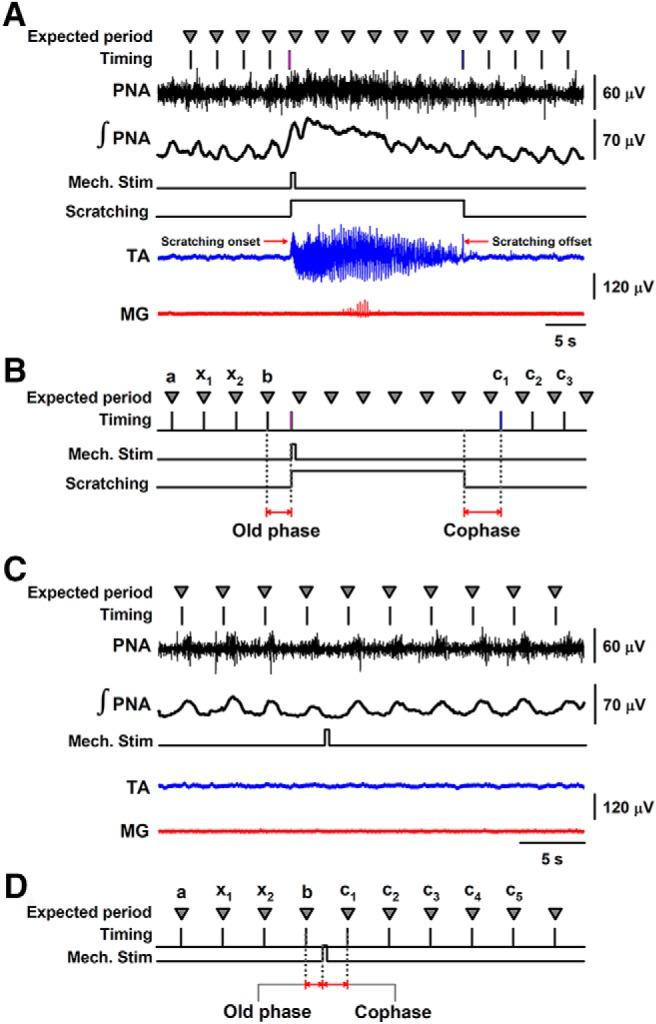
Scheme of the experimental paradigm. The phrenic nerve activity (PNA) was used to examine the respiratory rhythm. ***A***, A mechanical stimulus (timing indicated with a short rectangle) applied to the pinna elicits a fictive scratching episode (timing indicated with a large rectangle and the TA nerve activity). Such a stimulus was randomly applied after the fourth phrenic nerve discharge. During the scratching episode, there is an increase in the phrenic nerve activity [note the increase in the rectified and integrated PNA activity]. The periodic respiratory rhythm returns after the scratching episode ends. ***B***, A zoom of the timing scheme in ***A*** illustrating how the old phase and cophase were measured. The horizontal red arrows illustrate how the old phase and cophase were measured. ***C***, ***D***, The same as ***A***, ***B*** but for those mechanical stimuli that did not produce fictive scratching. The triangles illustrate the expected period of the PNA. The vertical black lines represent the experimental PNA. The pink and blue vertical lines indicate the onset and offset of the scratching episode, respectively. The rectangle labeled as “Mech. Stim” illustrates the phase in which the brief mechanical stimulation to the pinna was applied. The rectangle labeled as “Scratching” indicates the duration of the scratching episode.

We applied mechanical stimuli to the pinna randomly during the inspiration-expiration period. Each scratching episode was preceded by four or five control breaths, during which blood pressure and neural respiratory output remained constant. Following each fictive scratching episode, other additional four to eight breaths were recorded. The mechanical stimulus that produced fictive scratching was applied at various random times after the inspiratory phrenic burst (Fig. 1*A*,*B*).

We obtained graphs to examine phase resetting as described by Paydarfar and Eldridge (1987) and Winfree (1987). According to Winfree (1987), the “old phase” is the phase of the rhythm at which the stimulus begins and the “new phase (or cophase)” is the phase of the shifted rhythm extrapolated back to the moment the stimulus ends. We adapted these concepts to our experimental paradigm. We defined the old phase as the phase of the inspiratory rhythm at which the fictive scratching episode begins (Fig. 1*B*). The cophase was defined as the phase of the shifted inspiratory rhythm extrapolated back to the moment the scratching episode ends (Fig. 1*B*). Old phase and cophase are in cycle units, i.e., 1 is the period of the control cycle before stimulation. Our definitions of old phase and cophase are also consistent with Paydarfar et al. (1986, 1998) and Paydarfar and Eldridge (1987).

(1) Old phase =(time at scratching onset − b)/period

(2) Cophase i =(ci − time at scratching offset)/period

(3) Period⁢= (b−a)/N

**Figure 2.**
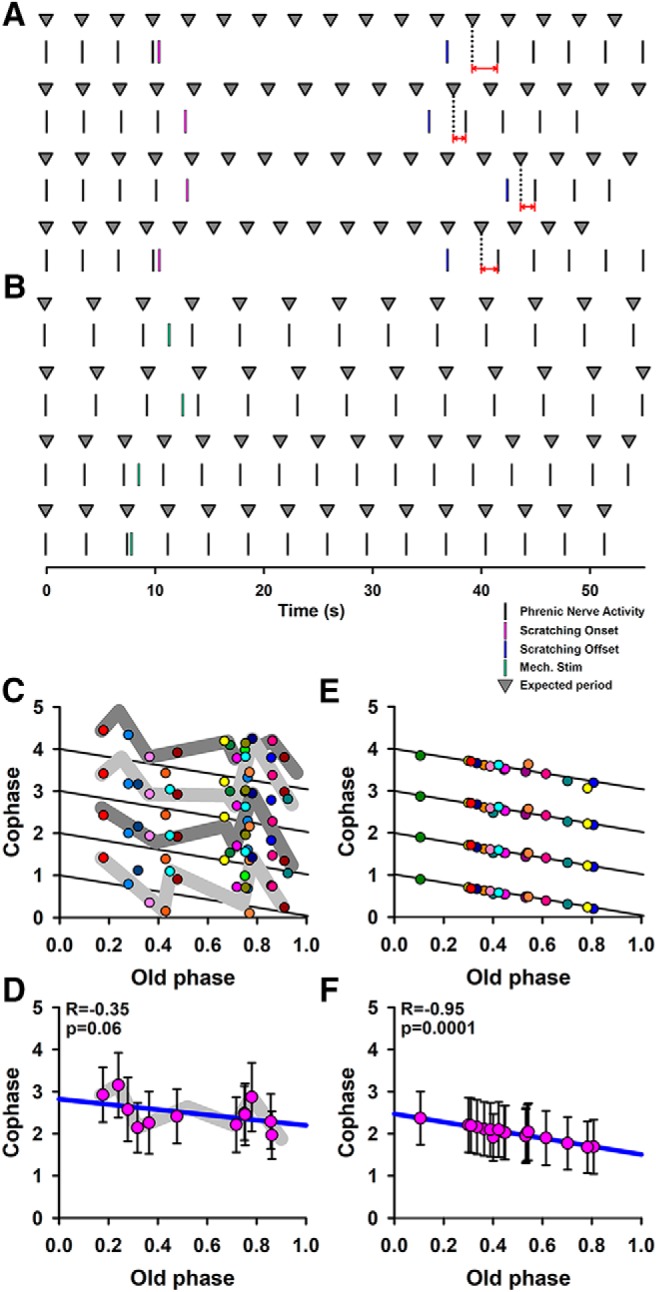
Phase analysis performed from recordings of phrenic nerve activity. ***A***, The vertical black marks indicate the phrenic nerve activity before, during, and after the application of mechanical stimuli applied to the pinna able to produce fictive scratching. The magenta and blue marks represent the onset and offset of the scratching episode. The triangles represent the expected period for the respiratory rhythmicity. The horizontal red arrows show the phase shift, which is the difference in phase in the respiratory rhythm as a consequence of the occurrence of the fictive scratching episode. ***B***, The same as ***A*** but for those mechanical stimuli not producing fictive scratching. The green marks represent the brief mechanical stimuli. ***C***, Phase-transition graph, constructed by the relationship between the normalized old phase and cophase. Note the tendency of the points toward a horizontal arrangement (i.e., type 0 resetting). Each color is related to each scratching episode that produced a phase shift of the respiratory rhythm. ***D***, Pink circles are the averaged data from ***C***, and the vertical lines are the standard deviation. ***E***, The same as ***C*** but for those mechanical stimuli not producing fictive scratching. Each color represents the trials in which the mechanical stimulation to the pinna was unable to change the phase. Note that in this case phase resetting did not occur and all points are arranged on the black lines following the expected period. ***F***, Averaged data from ***E***. The blue lines in ***D***, ***F*** represent the linear regression. The colors in ***A***, ***B*** are not related to the colors in the other graphs. The diagonal black lines (cophase = 1, old phase) depict the ideal case in which there is not a phase shift produced by the stimuli. A similar description of these diagonal lines was given by Winfree (1987). R indicates the correlation coefficient between old phase and cophase.

**Figure 3.**
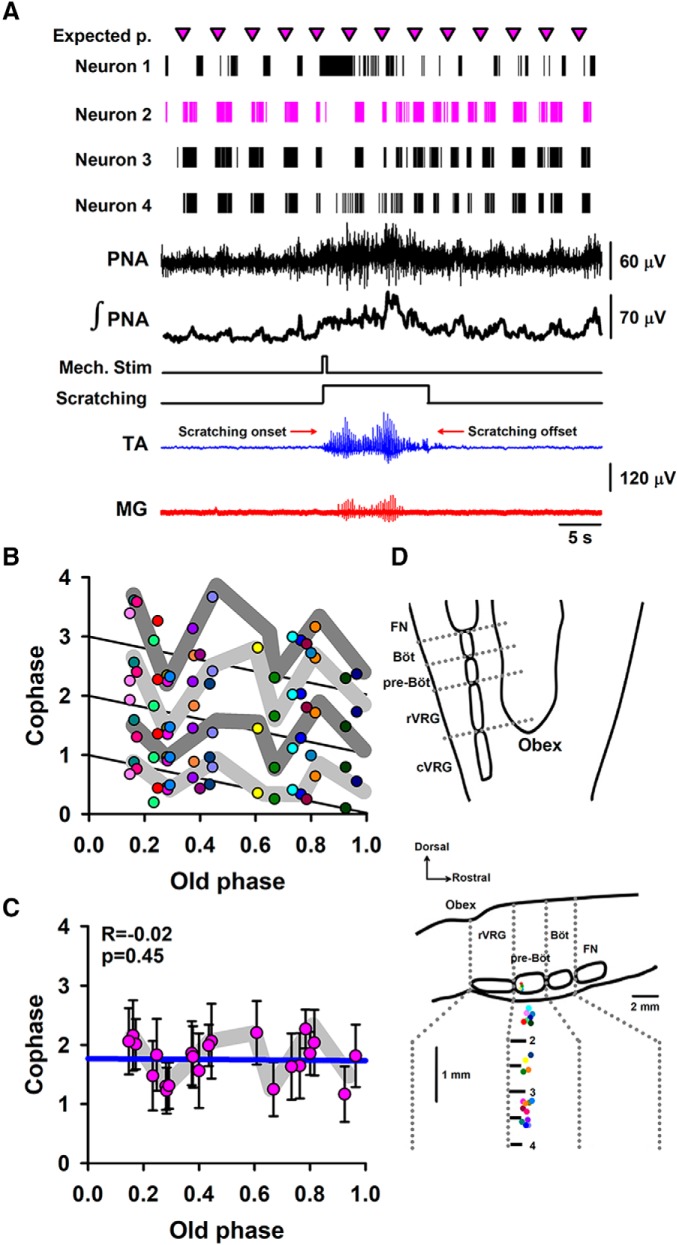
Phase analysis performed on the unitary neuronal activity recorded from the medulla oblongata. ***A***, The same format as Figure 1*A* but for the firing activity of four medulla oblongata neurons recorded simultaneously with the phrenic nerve activity (PNA). Pink triangles indicate the expected period for the bursting firing of neuron 2. ***B***, Phase-transition graph, constructed with the relationship between the normalized old phase and normalized cophase for the rhythmical firing activity of 22 neurons from the medulla oblongata, as a consequence of the occurrence of fictive scratching episodes. Each color in ***B*** is related to each interneuron that produced a phase shift of the respiratory rhythm. In ***D***, each color represents the location of each recorded interneuron. Note the tendency of the points toward a horizontal arrangement (i.e., type 0 resetting). The diagonal black lines (cophase = 1, old phase) depicts the ideal case in which there is not a phase shift produced by the stimuli. ***C***, Pink circles are the averaged data from ***B***, and the vertical lines are the standard deviation. The blue line in ***C*** represents the linear regression. ***D***, Schematic drawing of recording sites of rhythmical neurons from the medulla oblongata. R indicates the correlation coefficient between old phase and cophase. FN: Facial Nucleus, Böt: Bötzinger Complex, pre-Böt: pre-Bötzinger Complex, rVRG: rostral Ventral Respiratory Group, cVRG: caudal Ventral Respiratory Group.

Where i = 1–4 is the number of phrenic nerve bursts after scratching, and N is the number of phrenic nerve bursts before scratching (Fig. 1*A*,*B*). We calculated up to four successive cophases (i = 1–4) to compare our Figures 2*C*, 3*B* with the figures in the articles by Paydarfar et al. (1986, 1998) and Paydarfar and Eldridge (1987), who also used four cophases to illustrate the phase resetting.

These definitions of the old phase and cophase are consistent with the concepts stated by Paydarfar and Eldridge (1987) and Winfree (1987). The phase transition graph was obtained by constructing a plot of old phase versus the cophase (for review, see Canavier, 2015). The onset of inspiration was represented by the time when rectified, and integrated phrenic nerve activity increased to a level that was twice the baseline noise level (Paydarfar and Eldridge, 1987). The old phase and cophase values were expressed as a normalized fraction of the average period of four or five control breaths before the onset of the scratching episode. For example, in the case of the four control breaths illustrated in Fig. 1*B*, the period was calculated with Equation 3.

As a control, we also obtained graphs of cophase versus old phase for the respiratory rhythmicity associated with mechanical stimulation to the pinna that was unable to produce fictive scratching (Fig. 1*C*).

We defined the old phase as the phase of the inspiratory rhythm at which the mechanical stimulus to the pinna begins (Fig. 1*D*). The cophase was defined as the phase of the shifted inspiratory rhythm extrapolated back to the moment the mechanical stimulus ends (Fig. 1*D*). Old phase and cophase are in cycle units, i.e., 1 is the period of the control cycle before stimulation.

(4) Old phase =(time at mechanical stimulus − b)/period

(5) Cophase i =(ci − time⁢ at mechanical stimulus)/period

(6) Period⁢= (b−a)/N

Where i = 1–4 is the number of phrenic nerve bursts after “the mechanical stimulus applied to the pinna,” and N is the number of phrenic nerve bursts before the mechanical stimulus applied to the pinna (Fig. 1*C*,*D*).

## Results

We applied brief mechanical stimulation to the pinna to elicit fictive scratching episodes. As illustrated in Figure 1*A*, we applied such mechanical stimuli during the inspiration-expiration period. This allowed us to examine changes in the phase of respiratory rhythmicity related to the occurrence of these scratching episodes. The scratching episodes elicited by the brief mechanical stimulation lasted from ∼10 to 25 s. In most of the cases (21 of 25 episodes), the phrenic nerve exhibited an increase of firing activity associated with the occurrence of each scratching episode. Therefore, we only included in the analysis those scratching episodes associated with a phrenic nerve excitation. In 21 of 21 episodes, the phrenic nerve excitation during the scratching episode lasted the whole duration of scratching (Fig. 1*A*).

For the phase analysis, we measured the timing of phrenic nerve activity relative to the timing of onset and offset of fictive scratching. Figure 2*A* illustrates some of such timing marks for one animal. The marks in black color indicate the beginning of each rectified and integrated burst of phrenic nerve activity, whereas the marks in magenta and blue color indicate the onset and offset of fictive scratching, respectively. The triangles in Figure 2 represent the expected periodic activity of the phrenic nerve, which was calculated as the average of four or five periods of phrenic nerve activity before every scratching episode. We can note in Figure 2*A* the clear phase-shift in phrenic nerve activity after the extinction of every scratching episode (see horizontal red arrows). This phase-shift qualitatively indicated the occurrence of a resetting behavior in the phrenic nerve rhythm associated with the occurrence of scratching episodes. Therefore, we proceeded with the quantitative analysis of phase resetting using the Equations 1–3. The old normalized phase was calculated as “(old phase)/period” and the normalized cophase as “(cophase)/period.” In Figure 2*C*, we show a phase-transition graph of the “normalized old phase” versus “normalized cophase” for phrenic nerve activity resulting from the occurrence of scratching episodes from four cats. This graph shows the occurrence of a behavior with a tendency to type 0 resetting. Note how the gray shadows in Figure 2*C* follow a horizontal tendency, besides the variability in the cophase values.

We also analyzed the effects of mechanical stimulation of the pinna (that did not produce fictive scratching) on the phase of the respiratory rhythm for the same animals. This type of analysis served as a control for our observations of phase resetting associated with fictive scratching. Figure 2*B* shows that such mechanical stimulation to the pinna did not produce a phase shift in the respiratory rhythmicity. Note that the expected period was maintained after the mechanical stimulation. Therefore, we proceeded with the quantitative analysis using the Equations 4–6. Figure 2*E* shows a phase transition graph of the normalized old phase versus normalized cophase for the phrenic activity associated with this type of mechanical stimulation. We found that there was not a phase resetting of the respiratory rhythm for those mechanical stimuli to the pinna that did not produce fictive scratching. Note that none of the points in Figure 2*E* escaped from the expected black lines for a normal rhythm without phase shift. Figure 2*D*,*F* shows averaged data from Figure 2*C*,*E*, respectively.

To analyze in more detail the effects of fictive scratching episodes on the respiratory rhythmicity we analyzed the unitary firing activity of neurons from the medulla oblongata of two cats. We found 22 neurons whose rhythmic firing was synchronized with the phrenic nerve activity. These neurons changed their firing rhythmicity during the occurrence of the scratching episodes. Figure 3*A* shows the simultaneous recording of four of these neurons during a scratching episode. Note the synchrony between the rhythmic phrenic nerve activity and the interneuron bursting before scratching. It is clear that during scratching some neurons increase their firing rate becoming tonic, while other neurons decrease their firing activity. In general, during scratching the firing activity of neurons from the medulla oblongata becomes disorganized. When the fictive scratching episode subsides, the periodic burst of activity of the neurons is recovered but with a phase shift.

In Figure 3*B*, we show a phase-transition graph of the normalized old phase versus normalized cophase for the interneuronal activity resulting from the occurrence of scratching episodes from two cats. The methods to obtain this graph were similar to those followed for the phase transitions graphs for the phrenic nerve activity. We used similar formulas as those described in Equations 1–3 to calculate the normalized old phase and normalized cophase for the neuronal firing. We found a clear phase shift for all the neurons. Figure 3*A* shows in pink traces the phase shift for neuron 2. The graph in Figure 3*B* shows the occurrence of a behavior with a tendency from type 1 to type 0 resetting. This finding indicates that there are neurons in the medulla oblongata which exhibit phase resetting associated with the activation of the scratching CPG. Figure 3*C* shows averaged data from Figure 3*B*.

**Figure 4.**
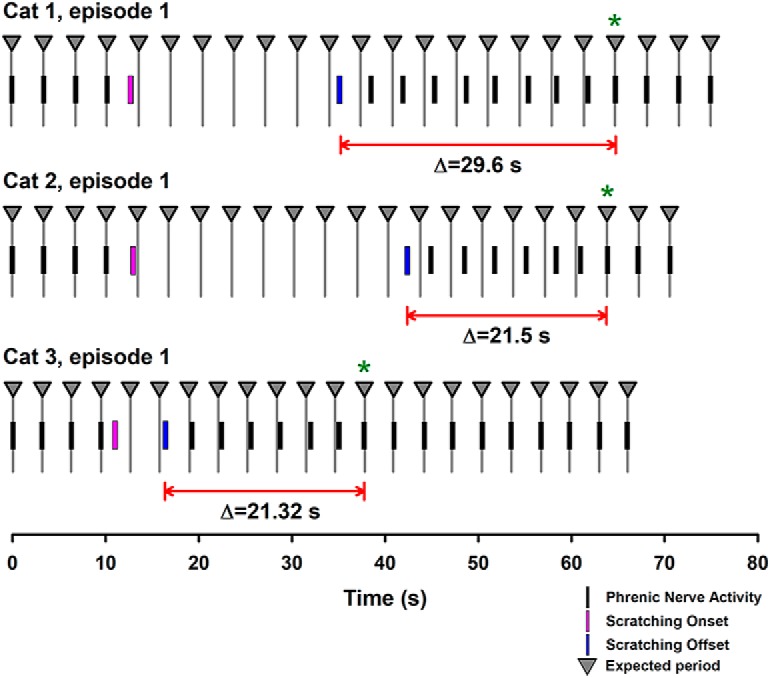
The timing of the phrenic nerve activity before and after scratching episodes in three cats. The black vertical lines illustrate the phase shift in the phrenic nerve activity after the scratching offset and its return (see asterisk) to the expected rhythm. Such return last 29.6, 21.5, and 21.32 s for these animals.

**Figure 5.**
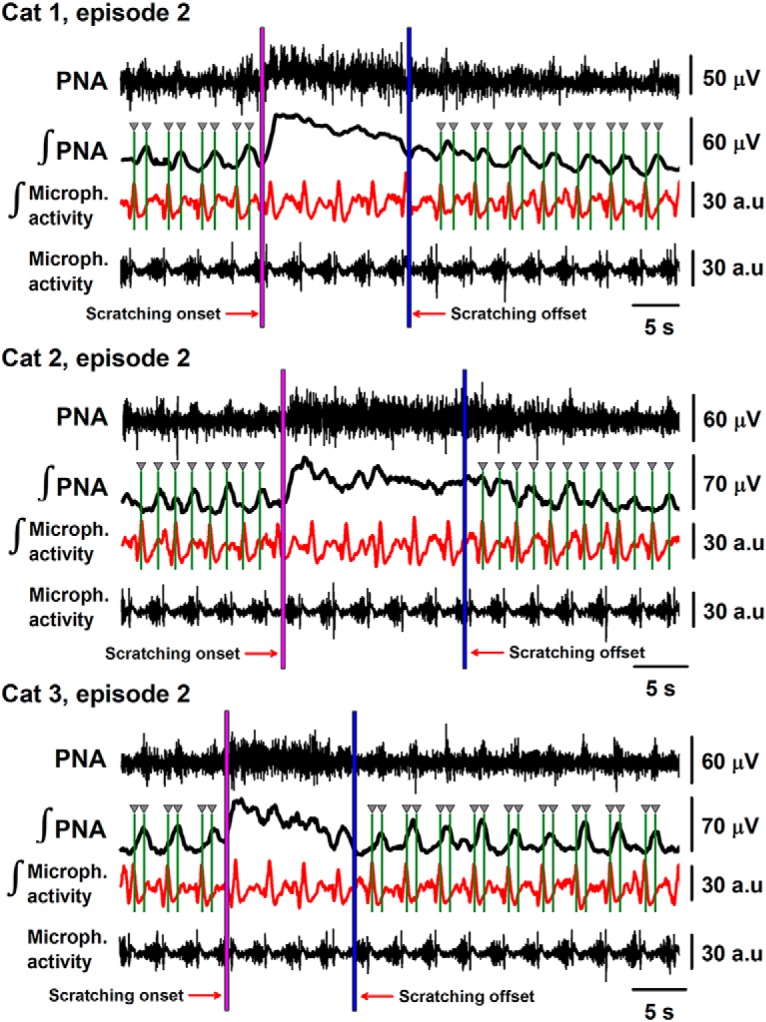
The timing of phrenic nerve activity and respiratory rhythm imposed by artificial ventilation in three cats as indicated. First trace, phrenic nerve activity (PNA). Second trace, Rectified and integrated phrenic nerve activity. Third trace, Rectified and integrated sound signals recorded with an electret microphone (red traces). Fourth trace, Sound signals (microphone activity, i.e., respiratory activity recorded with an electret microphone). The vertical green lines illustrate the timing between the inspiratory activity and the maximal peak of the rectified and integrated phrenic nerve activity. The beginning of the scratching episode is indicated with the pink line (scratching onset). The end of the scratching episode is illustrated with the blue line (scratching offset). There is the same time separation between the vertical green lines before and after the scratching episode.

We analyzed in more detail the phrenic nerve activity during a more extended period after the scratching episodes. We found that the activity of the phrenic nerve was not synchronized with respiration during 22.8 ± 2.7 s after the end of the scratching episode. This means that after that time the phrenic nerve becomes synchronized again with the artificial respiratory rhythm (see examples for three cats in Fig. 4). In Figure 5, we show the rectified and integrated signal from an electret microphone sensing respiratory sounds in three cats (red traces, inspiration upwards). Note that before the onset of scratching, the maximum peak of the rectified and integrated phrenic nerve activity is phase synchronized with the imposed respiratory rhythm (see green vertical lines). Furthermore, there is a clear phase shift in the rectified and integrated phrenic nerve activity after the scratching offset (see how the maximal peak of the phrenic nerve activity is displaced on the left, relative to the right green lines). However, ∼22.8 ± 2.7 s after the scratching offset, the phrenic nerve activity becomes phase synchronized again with the artificial respiratory rhythm.

## Discussion

We found that those stimuli to the pinna that did not elicit fictive scratching did not reset the respiratory rhythm. However, when the pinna stimuli elicited fictive scratching, then the respiratory rhythm exhibited a significant phase resetting. Our results support the findings by Paydarfar et al. (1986), Paydarfar and Eldridge (1987), and Eldridge et al. (1989), who demonstrated for the first time that a phase resetting exists for the neural oscillator that controls breathing.

In 1931, King studied the effect of respiration on the scratch reflex in two dogs and one cat. The scratch movements were registered using a pulley and lever system, whereas the respiration was recorded by a pneumograph and tambour system, or by a compound level and pulley system. He obtained two findings. First, there is a lowering of the scratching threshold during inspiration; and second, the after-discharge may reappear during inspiration for several respiratory cycles. These results were consistent with previous observations from the same author, who reported that there is an inspiratory augmentation of proprioceptive reflexes. He concluded that the scratch reflex is affected by the respiratory activity, assuming that impulses from the respiratory center contribute to the excitatory state in the spinal cord, which in turn is manifested by a lowering of the threshold and by an increase in the number of active motoneurons (King, 1931). However, to our knowledge, there are no reports in the literature examining the resetting effects of the scratching CPG on the respiratory rhythm. In this context, our study is original and contributes to revealing that there is a resetting in the interaction between the scratching and respiratory CPGs.

Paydarfar et al. (1986) studied resetting patterns resulting from electrical stimuli of graded intensity applied to the superior laryngeal nerve in anesthetized paralyzed cats. They found results ranging from attenuated stimuli giving type 1 resetting to strong stimuli resulting in type 0 resetting. Based on this finding, we can acknowledge that a limitation of our study is the difficulty to control the intensity and duration of the scratching episodes. Therefore, in our experiments, it is not possible to examine such transition from type 1 to type 0 resetting. However, besides this limitation, our results strongly suggest that there is phase resetting when the scratching CPG interacts with the respiratory CPG. The first argument in support of this statement is that our graph shown in Figure 2*C* tends to type 0 resetting (although with high variability). Paydarfar et al. (1986), Paydarfar and Eldridge (1987), and Eldridge et al. (1989) reported the type 0 resetting. A second argument is that Paydarfar and Eldridge (1987) found that the electrical stimulation of the midbrain reticular formation and periaqueductal gray matter can evoke facilitation of phrenic nerve activity in the cat. This is consistent with our observation that the activation of the scratching CPG, which in turn is related to an activation of the bulbar reticular formation (Tapia et al., 2013), also produces facilitation in the activity of the phrenic nerve (Fig. 1*A*).

On the other hand, the phase resetting has also been useful to advance in the comprehension of the neural architecture of the CPGs for locomotion and scratching. Lafreniere-Roula and McCrea (2005) used phase resetting as a parameter of interaction among both pattern generators to demonstrate that the same neural substrate is shared by the pattern formation layer of the CPGs for both locomotion and scratching. Therefore, our findings also allow us to suggest the existence of shared neural substrates in the neural architecture of the respiratory and scratching CPGs.

A necessary implication of our study is that the scratching CPG can reset the respiratory CPG even though the cats were paralyzed, thus showing that the central interaction between CPGs could be enough to allow phase resetting and entrainment. This means that the muscle machinery during the “motor action” could not be required to guarantee phase resetting in the interaction between both oscillators. Such reasoning is consistent with several observations made in *in vivo* and *in vitro* preparations in the context of locomotion. The first observation is that the respiration rate in cats can increase during fictive locomotion despite the absence of muscular contraction or limb movements (Eldridge et al., 1985; Le Gal et al., 2014). The second observation is that in the isolated neonatal rat brainstem-spinal cord preparation the increase in respiratory rate observed during fictive locomotion is associated with an increase in excitability of pre-inspiratory neurons of the parafacial respiratory group (Le Gal et al., 2014). The third observation is that the respiration rate increases following stimulation of the mesencephalic locomotor region in an *in vitro* lamprey preparation, even in the absence of the spinal cord and caudal brainstem (Gariépy et al., 2012; Missaghi et al., 2016). The fourth observation is that the respiratory drive in hindlimb motoneurons in decerebrate cats with neuromuscular blockade is transmitted via elements of the locomotor CPG (Wienecke et al., 2015). This is also in line with the “central command hypothesis” stipulating that central neural connections activate the respiratory centers during exercise to regulate a safe exertion by the body (for review, see Gariépy et al., 2010).

Coupled microcircuits generate respiration, as the pre-Bötzinger complex (pre-BötC), which is composed of a master clock of excitatory rhythmogenic neurons and pattern-formation networks of excitatory and inhibitory interneurons that in concert produce the strong periodic drive for inspiration (for review, see Del Negro et al., 2018). Because the pre-BötC coordinates all phases of the breathing cycle and it is influenced by many inputs including emotion and cognition (Del Negro et al., 2018), it is comprehensible that the CPG circuit for scratching also could produce an effect on it. Our finding of phase resetting of interneurons from the medulla oblongata (near pre-BötC and the rostral ventral respiratory group rVRG) associated with the scratching episodes strongly supports this possibility.

It is important to mention that our main finding is not to show the synchrony between brainstem neurons and phrenic nerve activity. Our main finding is that in the brainstem respiratory centers there are neurons exhibiting phase resetting associated with the activation of the scratching CPG. This is a relevant contribution to the understanding of the resetting action of the spinal CPG on the brainstem respiratory CPG.

We conclude that fictive scratching, as a “central stimulus” delivered to the respiratory CPG, can produce phase resetting in phrenic nerve activity and the firing activity of interneurons from the medulla oblongata in decerebrate/paralyzed cats.

## Synthesis

Reviewing Editor: Elizabeth Cropper, Mt Sinai Sch Med Decisions are customarily a result of the Reviewing Editor and the peer reviewers coming together and discussing their recommendations until a consensus is reached. When revisions are invited, a fact-based synthesis statement explaining their decision and outlining what is needed to prepare a revision will be listed below. Note: If this manuscript was transferred from JNeurosci and a decision was made to accept the manuscript without peer review, a brief statement to this effect will instead be what is listed below.

This manuscript was transferred from the Journal of Neuroscience. The previous reviewers felt that the data are of high quality and that conclusions are supported by data. However, they felt that the findings were not novel enough for the JN. The authors revised the manuscript and addressed all the previous comments including the issue of significance.
